# Intention Estimation Using Set of Reference Trajectories as Behaviour Model

**DOI:** 10.3390/s18124423

**Published:** 2018-12-14

**Authors:** Naveed Muhammad, Björn Åstrand

**Affiliations:** School of Information Technology, Halmstad University, 30118 Halmstad, Sweden; bjorn.astrand@hh.se

**Keywords:** behaviour modelling, intention estimation

## Abstract

Autonomous robotic systems operating in the vicinity of other agents, such as humans, manually driven vehicles and other robots, can model the behaviour and estimate intentions of the other agents to enhance efficiency of their operation, while preserving safety. We propose a data-driven approach to model the behaviour of other agents, which is based on a set of trajectories navigated by other agents. Then, to evaluate the proposed behaviour modelling approach, we propose and compare two methods for agent intention estimation based on: (i) particle filtering; and (ii) decision trees. The proposed methods were validated using three datasets that consist of real-world bicycle and car trajectories in two different scenarios, at a roundabout and at a t-junction with a pedestrian crossing. The results validate the utility of the data-driven behaviour model, and show that decision-tree based intention estimation works better on a binary-class problem, whereas the particle-filter based technique performs better on a multi-class problem, such as the roundabout, where the method yielded an average gain of 14.88 m for correct intention estimation locations compared to the decision-tree based method.

## 1. Introduction

Autonomy in a mobile robotics context refers to the operation of a robot without any human-operator intervention. While it is possible that an autonomous mobile robot might have to operate in a rather solitary setting, very often, robot operating environments are populated with other agents. These agents can be other robots, manually driven vehicles, humans, etc. One such example is an autonomous forklift truck on a factory or warehouse floor, which has to operate in the presence of other forklift trucks as well as humans. Another example is autonomous cars that are expected to drive safely in the presence of other cars, bicycles, pedestrians, etc.

Autonomous nature of such robots makes it imperative for them to be able to model the behaviour and estimate the intentions of other agents to enhance efficiency, without compromising on safety (their own, as well as that of the other agents). For instance, an autonomous car or forklift truck might be able to guarantee safety using only its emergency-braking feature (by reducing its nominal speed every time anything comes close to it), but this is undesirable for a number of reasons. First, in doing so, its time to perform an operation might increase beyond a meaningful limit. Secondly, using only an emergency breaking layer for safety will result in a non-smooth drive resulting in lack of comfort, as well as high wear and tear of multiple mechanical elements in a vehicle. Therefore, a higher-level understanding of an autonomous vehicle’s environment is needed so that the vehicle navigation system acts in a situation-aware manner and anticipates (well before the emergency brakes need to be activated) the intentions of agents present in its environment. As humans (while driving a car or operating a forklift truck), we make decisions based on our understanding and perception of others’ behaviour all the time. A detailed discussion on open issues concerning pedestrians and autonomous cars, and how heavily the safety and efficiency on roads is based on the mutual understanding of behaviour between humans (be it vehicle drivers or pedestrians), is presented in [1].

Agent behaviour modelling in autonomous robotics and transportation context has been investigated extensively in the literature. Pedestrian intentions are estimated using dynamic fuzzy automa, employing pedestrian heading angle, velocity and position offset from curbs in [2]. A study on estimation of driver intention on roads that uses a combination of fuzzy logic for turning at intersections and Gipps model for car following is presented in [3]. In the study, the road geometry at intersections is modelled using links joined in different sequences in the form of a graph. A study on driver intention from the perspective of road safety is presented in [4], where driver behaviour is modelled using artificial potential fields. Deep learning has also been employed in studies addressing driving behaviours. A recent study [5] presents clustering of similar driving encounters (among five different types of encounters) using auto encoder and GPS trajectories of vehicles. A model to infer driver intention at turns as well as while following other vehicles, termed intelligent driver model, is proposed in [6]. A survey of studies addressing drive style recognition is presented in [7].

Prediction of vehicle behaviour using Hidden Markov Models (HMM) is investigated in several studies that address the subjects such as ADAS (Advanced Driver Assistant Systems) and safety. For example, Jain et al. [8] presented a study that employs multiple sensing sources including cameras (inside a vehicle to capture driver’s face and outside the vehicle to capture traffic context), GPS and street maps, to predict car future manoeuvres (such as lane change, turning and going straight). The application area of Jain et al. [8] is ADAS. HMM is employed by Maghsood and Johannesson [9], using on-board vehicle data (available in most vehicles via CAN bus) to detect turning right or left and going straight events. Another study that addresses the assessment of threats (to ego vehicle) caused by other vehicles using HMM and random forests is presented in [10]. It presents results on simulation data that validate the proposed technique.

Some works address only the modelling of acceleration and deceleration behaviour of vehicles in traffic with applications in safety in autonomous driving (among other application areas such as intersection design and traffic simulation, etc.). For instance, Bokare and Maurya [11] investigated acceleration and deceleration of different vehicle types. Similarly, Maurya and Bokare [12] investigated deceleration behaviour among different vehicle types.

Similarly, some studies investigate only the lane-change behaviour in traffic. A system to aid lane changes that uses optical-flow on images, captured by a camera placed on rear-view mirror, is presented in [13]. Another work that employs vision sensors for predicting lane-changes in traffic is presented in [14].

A method to estimate other vehicles’ intentions in a merging scenario, based on probabilistic graphical model, is presented in [15]. A method that provides reachable paths that an agent on road might take is presented in [16]. The study presents conditional transition maps that provide which paths, from any given location, is reachable, without explicitly providing probabilities for all reachable paths. We propose a method for modelling agent behaviour which is based on stored raw trajectories taken by a category of agents in a physical space or environment. Our approach, thus, does not create an overall conditional probability graph, but instead is based on a set of agent trajectories as behaviour model.

Many studies, in the context of autonomy on road, have proven machine learning techniques such as support vector machines (SVM) and neural networks (NN) to work well for agent intention estimation. Intention estimation in a car-following scenario is investigated using SVM, NN, and HMM in [17]. Driver intention estimation in lane-change scenario, based on SVM, is presented in [18]. A study presented in [19] also employs SVM, and addresses vehicle behaviour modelling in roundabouts with the aim of predicting vehicle behaviour (in terms of staying inside or exiting the roundabout) as early as possible. The study employs GPS, steering angle sensor and odometer for data acquisition, and uses steering angle as a feature attribute. Our model also uses absolute heading angle as one of the feature attributes but (in contrast to Zhao et al. [19] who employed on-board sensors), in our study, one of the datasets (belonging to a roundabout) is based on trajectories extracted from perception data acquired using a lidar sensor placed at the center of a roundabout. A similar investigation, presented in [20], uses data from a driving simulator to model driving behaviour. The aim of the study is to investigate the impact of roundabout layout on driving behaviour. In contrast to Zhao et al. [20], our study employed real-world datasets from agent behaviour modelling and intention estimation. The contributions of this paper are described in Section 1.1.

A survey of detection, tracking and behaviour analysis in traffic is presented [21]. It comprehensively surveys vision-based techniques employing different sensor types (monocular, stereo, etc.), mathematical models, features, benchmarks, etc.

### 1.1. Contributions

The contributions of this paper are as follows. First, we propose a non-parametric, data-driven way of modelling the behaviour of agents. Secondly, we propose and compare a filtering and a machine-learning based approach for agent intention estimation. The comparison of the two techniques presented in this paper shows that, overall, the machine-learning based approach performs better for a binary-class problem, whereas the filtering based approach performs better for a multi-class problem. It is worth mentioning here that our data-driven modelling approach does not require huge amounts of data (but instead, a small set of reference trajectories).

### 1.2. Structure of the Paper

The remainder of this paper is structured as follows. Section 2 presents the methodology for behaviour modelling as well as intention estimation using two methods. Section 3 first introduces the datasets used to validate the proposed approaches, and then presents the experimental results on the datasets. A discussion on the results obtained, as well as the usability of the proposed approaches, is presented in Section 4. The paper concludes in Section 5.

## 2. Method

We propose a data-driven approach for agent behaviour modelling. The behaviour model takes a wisdom-of-the-crowd approach and models agent behaviour based on set of trajectories (and feature values) that agents belonging to a specific category traversed. The proposed behaviour modelling approach was evaluated using agent intention estimation. Two methods for intention estimation are proposed in this paper, which are based on: (i) particle filtering; and (ii) decision trees. The subsections below describe the proposed behaviour modelling layer, the particle-filter and the decision-tree based intention estimation algorithms, sequentially.

### 2.1. Behaviour Model

**Figure 1:**
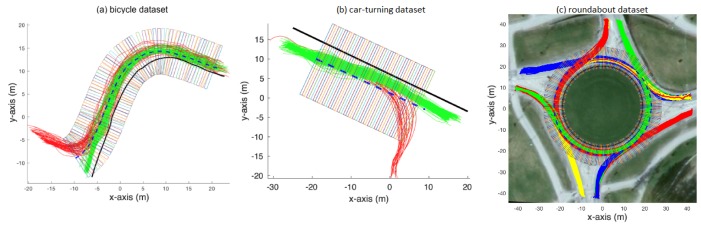
Trajectories and the discretisation grid for: (**a**) bicycle dataset; (**b**) car-turning dataset; and (**c**) roundabout dataset.

**Figure 2:**
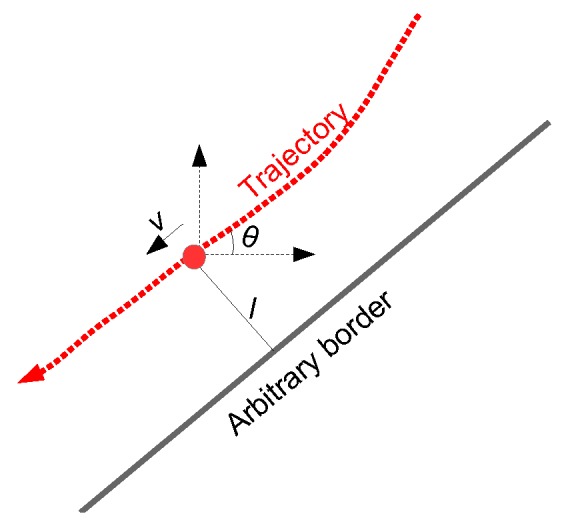
Attribute definitions: For a given position on a trajectory (trajectory represented by the dashed red line and a given position by the red dot), θ is absolute angle in degrees, *l* is the shortest distance from the border of the path (which can be arbitrarily drawn), and *v* is speed along the trajectory.

The proposed behaviour model is non-parametric and is based on a set of reference trajectories traversed by agents in a given environment. Figure 1a shows two types of bicycle trajectories on a bicycle track marked in red and green. Both types of trajectories originate from top-right of the figure but split into two paths towards the bottom-left of the figure. We propose the physical space to be discretised into a grid (with each cell having a width *w*) along the physical bicycle track. Such a discretisation grid is also shown in the figure, where w= 0.6 m. Physical-space based discretisation has also been employed by Zhao et al. [19] in their SVM-based intention estimation. In our proposed behaviour modelling layer, a set of *q* number of trajectories drawn from two categories of agent behaviours are taken as reference trajectories. Then, for each cell on the grid, different attributes for each of the reference trajectory are calculated including absolute heading angle θ, speed *v* and the lateral position offset *l* with reference to an arbitrarily drawn (or physical border of the bicycle track, in this case, if it is available) boundary. Definitions of the three attributes are presented in Figure 2. If more than one observations fall inside a grid cell, mean of the observed attribute values are assigned as attribute values to the corresponding grid cell. In this way, such a physical-space based discretisation grid has a twofold advantage. First, it allows for agent behaviour to be linked to physical location of an agent in an environment (instead of, for instance, how much time an agent has been inside an environment), and, second, it helps low-pass filter any noisy attribute observations. A behaviour model *B* can thus be represented as:

(1) B={θ,v,l∈IR}

**Figure 3:**
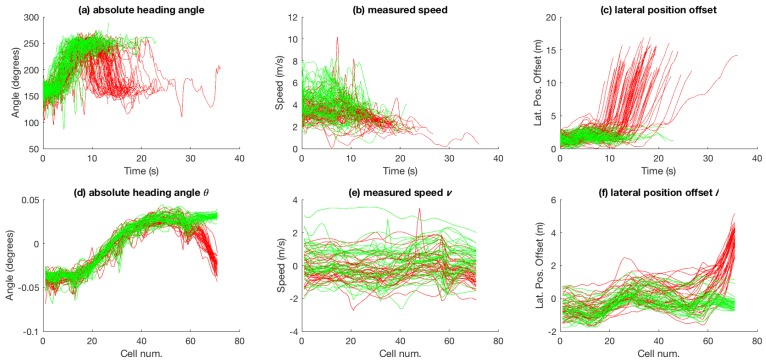
Attribute values for the bicycle dataset: (**a**–**c**) observations across time; and (**d**–**f**) grid-cell-wise zero-mean and unit-variance normalised attribute values.

The attributes for the trajectories shown in Figure 1a are presented in Figure 3. Figure 3a–c shows the attribute values measured in time and Figure 3d–f shows the attribute values after zero-mean and unit-variance normalisation plotted against the grid shown in Figure 1a.

### 2.2. Using Particle Filtering

Particle filters have extensively been used in the literature. An overview of the advances in particle filtering is presented in [22]. In robotics, particle filters have been used for tasks such as (but not limited to) map-based localisation, presented for instance in [23,24]. Two studies that provide tutorials on particle filtering methods are presented in [25,26]. Our particle-filter based algorithm for intention estimation is described below.

Our proposed algorithm takes a map provided by the behaviour modelling layer (cf. Section 2.1). This map essentially consists of physical grid locations in an environment of interest and the feature values that agents have taken (in terms of the respective reference trajectories used by the modelling layer). A feature here consists of one or more of the three attributes θ, *v* and *l* described in the previous subsection. The state, i.e., the category to which a query instance, at a discretised location (in a grid space) *d* belongs, is represented by the set of particles $X_{d}$, which consists of *M* particles.

(2) $$X_{d}=\left\{x_{d}^{\left[1\right]},x_{d}^{\left[2\right]}\ldots x_{d}^{\left[M\right]}\right\}$$

Each of the *M* particles in the set $X_{d}$ represents the query instance to belong to one of the several reference trajectories in the map (provided by the behaviour modelling layer). Each particle also has a weight $w_{d}^{\left[m\right]}$ associated to it. At the beginning, a particle set $X_{0}$ is randomly generated, where each particle $x_{0}^{\left[m\right]}$ contains a possible state. For instance, for a map provided by the behaviour modelling layer that contains ten reference trajectories forming the set *B* (q=10, i.e., five trajectories for each agent behaviour in a binary-behaviour case), each particle contains a randomly generated number between 1 and 10. Initial weights $w_{0}^{\left[m\right]}$ are all assigned the same value of 1/M, in other words, each particle is equally likely to be representing the actual state.

This initial particle set is then recursively updated, at each change of location in terms of *d*:

1. Make the observation $z_{d}$, i.e., measuring the feature value at current grid location of the query instance.
2. Calculate the weight factor for each particle depending on how consistent the current measurement $z_{d}$ is with each of map trajectories.
  (3) $$w_{d}^{m}=p\left(z_{d}|x_{d}^{m}\right)$$
  This is implemented by calculating the Euclidean distance between $z_{d}$ and the corresponding feature value in each of the map trajectories.
3. Draw, with replacement, *m* particles from the updated particle set, with probability equal to particles’ associated importance weights to create updated particle set $X_{d}$. Many alternative resampling methods also exist in the literature and an in-depth study on such methods is presented in [27].

At any given location of the query instance on the grid, the sum of probabilities of particles representing map trajectories in each of the two categories represents the belief of the query instance for belonging to that category of the possible agent behaviour, thus allowing our approach to employ particle filtering for classification. This implementation of a particle filter is inherently different from Kalman filtering, which operates in a recursive predict-and-then-update fashion.

### 2.3. Using Decision Trees

The decision-tree based intention estimation algorithm, employs the same map provided by the modelling layer as employed by the particle-filter algorithm. In the decision-tree based algorithm however, at each grid cell location, a local decision tree [28] is computed using the feature data for two or more agent behaviour categories. When a query instance comes in, at each grid location that the query agent reaches, the local decision tree at that grid location is used to estimate the intention of the query agent from the possible agent behaviour categories. Despite using multiple decision trees, our technique differs from random forests (e.g., [29,30,31]) in that in random forests a set of decision trees is used for one instance of decision making or classification, whereas in our approach only one decision tree is used in each grid cell.

### 2.4. Validation Method

The two proposed intention estimation techniques were validated using the three datasets described in detail in Section 3.1. The validation was done in an n-fold and leave-one-out cross validation way for the two first datasets (bicycle and car-turning datasets), as they have a limited number of samples (82 and 34 trajectories, respectively). For a given iteration of the n-fold testing, five feature sets were tested (for both the proposed methods of particle filtering and decision trees) L (θ,v,l), (θ,l), (*v*), (*l*), and (θ). Aggregate results (the term aggregate results is used throughout the text and refers to the results for experiments performed on a complete dataset to make a distinction from some instances where results are generated on a subset of a dataset (for example, in Section 3.2)) are generated using the average of n-fold and leave-one-out tests for a given estimation method and feature set.

The third dataset, i.e., the roundabout dataset, consists of 456 trajectories and therefore the n-fold or leave-one-out cross validation was not needed. Instead, a subset (randomly drawn half) of the dataset was used as reference trajectories and the remainder as test set.

## 3. Results

This section presents the datasets used and experiments performed for validation of the behaviour modelling and intention estimation techniques proposed in Section 2.

### 3.1. Data

Three datasets were used for the experimentation to validate and compare the proposed intention estimation methods.

The first two datasets, provided by Viscando Traffic Systems AB [32], are based on agent trajectories acquired using a stereo-vision system. The first dataset consists of two types of bicycle trajectories. The dataset is presented in Figure 1a and Figure 3. Figure 1a shows the raw trajectories of bicyclists that come from top-right and eventually split into one of the two direction towards bottom-left of the figure. Figure 3 shows the absolute heading angle, measured speed, and lateral position offset (with respect to an arbitrarily drawn border of the bicycle track represented by the black solid line in Figure 1a) plotted across time in Figure 3a–c. The dataset consists of 42 trajectories for each of the two categories of bicycle paths. Grid cell based attribute (θ, *v* and *l*) values for the dataset are shown in Figure 3d–f.

**Figure 4:**
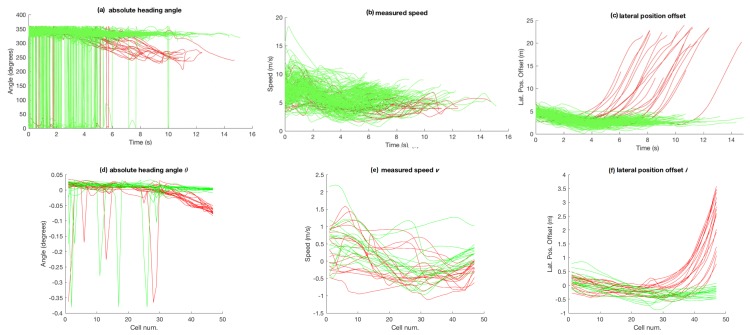
Attribute values for the car-turning dataset: (**a**–**c**) observations across time; and (**d**–**f**) grid-cell-wise zero-mean and unit-variance normalized attribute values.

The second dataset (among the two datasets provided by [32]), referred to as car-turning dataset in the sections that follow, consists of two types of car trajectories and is shown in Figure 1b. The figure shows the trajectories of cars that come from the left and either go straight or turn downwards. The dataset consists of 17 trajectories for each of the two categories. Grid cell based attribute (θ, *v* and *l*) values, after zero-mean and unit-variance normalisation, for the dataset are shown in Figure 4. Please note that the spikes in the angle data are believed to result from noisy position estimates. The noise was not explicitly removed from this dataset to check the robustness of the proposed techniques against data with such noise.

**Figure 5:**
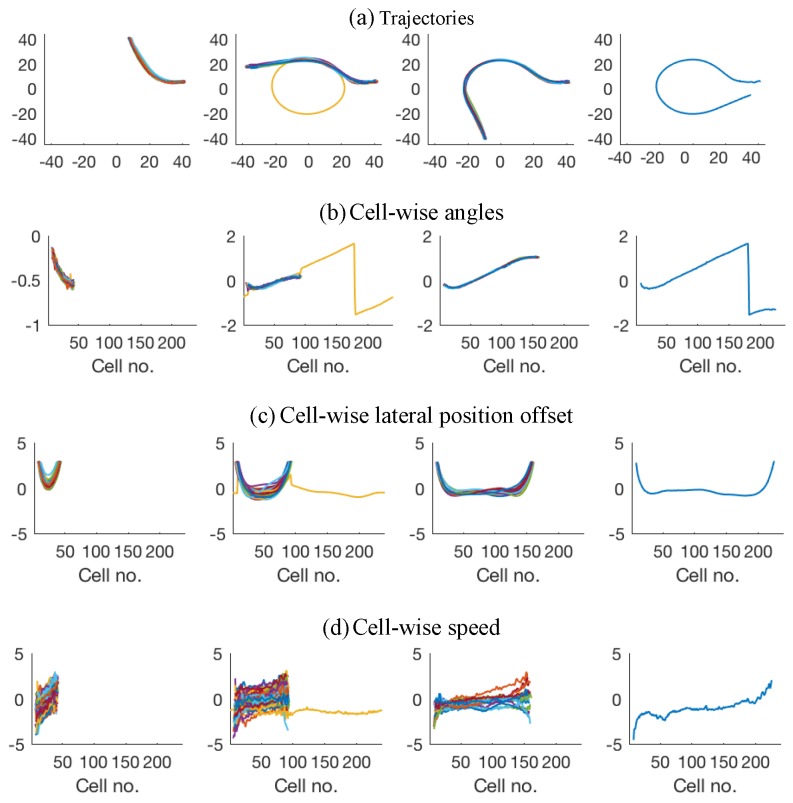
Trajectories and normalized attribute values for four categories corresponding to the East entrance in the roundabout dataset. Note that, only one u-turn trajectory exists (fourth column) in the whole dataset, which makes intuitive sense as vehicles rarely use roundabouts for performing u-turns compared to traffic exiting at first, second or third exits (with respect to any entry point). Similarly, second column of the figure includes one trajectory where a vehicle enters from East, and takes one complete circle around the roundabout, before finally exiting at the West.

The third dataset consists of traffic at a busy roundabout perceived using a multi-beam lidar sensor placed at the centre of the roundabout, originally acquired by Kucner et al. [16], Fan et al. [33], and is shown in Figure 1c. The discretisation grid shown in the figure has 240 cells (starting from cell No. 1 at $0^{\circ }$ degrees at positive x-axis, through cell No. 240 at $360^{\circ }$), with each cell having a size of 0.6 m by 10 m. The dataset contains 456 trajectories in total, for vehicles entering and exiting at the four (referred to as East, North, West and South) entry/exit locations of the roundabout. Figure 5a shows the trajectories for all the vehicles that enter from the East entrance and exit in either of the four directions (i.e., North, West, South or East) in the dataset. Figure 5b–d shows the normalised attribute values for corresponding to the each of the four categories of shown in Figure 5a. As vehicles entering from each of the four direction form four subsequent categories, the whole dataset is categorised into 16 categories, i.e., for vehicles entering from each direction and taking the first, second, third, or rarely even the fourth corresponding exit (i.e., rare occasions when vehicles use the roundabout for taking u-turns). Similarly, the dataset also contains a rarely occurring instance where a vehicle enters from the East, takes a complete circle around the roundabout and then exits in the West.

### 3.2. Basic Experiment Using Particle Filtering

**Figure 6:**
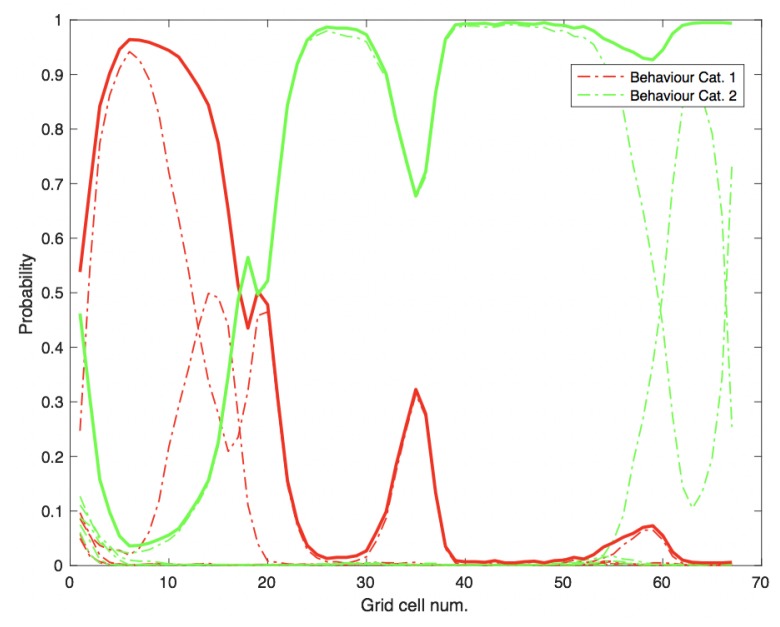
Evolution of beliefs, over the iterations of particle filter, on how belief for first or second category evolved.

In a basic experiment conducted using the particle-filter based intention estimation described in Section 2.2, five trajectories were randomly chosen from each of the two categories of the bicycle dataset (cf. Figure 1a) (i.e., q=10, forming the behaviour model *B*). Then, a trajectory from the remaining trajectories of category 2 (green) was chosen at random as query trajectory. The width of each grid cell *w* in this experiment was set to 0.6 m (which is also the case for all experiments presented in this paper, unless stated otherwise). The purpose of this basic experiment was to show how the proposed particle-filter based intention estimation method is used to classify a query trajectory to belong to one of multiple possible categories. The results of the experiment are presented in Figure 6. The figure shows how the belief of the query trajectory for belonging to one of the ten (five for each category) reference trajectory evolves (represented by dashed lines) as well as the sum of the beliefs for each category (represented by solid lines). Features used for this experiment contain angle θ, speed *v* and lateral position offset *l* information. The figure shows how the belief that the query instance belongs to category 2 (i.e., green) remains high from just after grid cell No. 20 until the end.

### 3.3. Aggregate Results Using Particle Filtering

**Figure 7:**
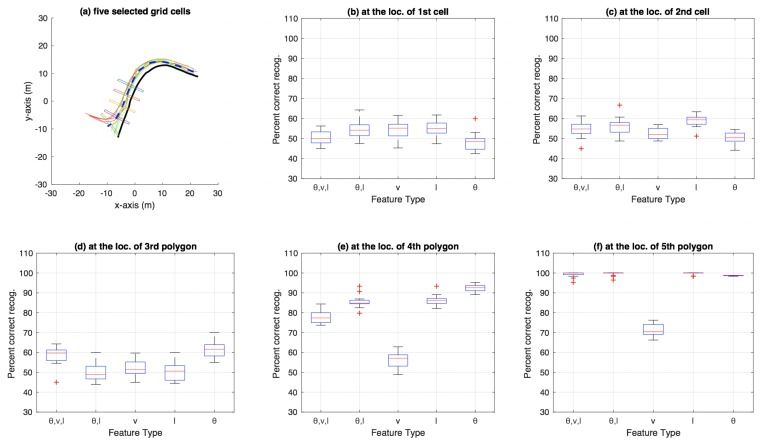
Aggregate results for bicycle dataset using particle filtering: (**a**) the five grid cells show locations where 60%, 70%, 80%, 90%, and 99% of the total bicycle path length (for the path discretised using the grid shown in Figure 1a) has been traversed; and (**b**–**f**) box plots at each of the five grid-cell locations in (**a**). Each subfigure (**b**–**f**) shows a box plot for percentage of correct category recognition for different feature types used including (θ,v,l), (θ,l), *v*, *l* and θ.

**Figure 8:**
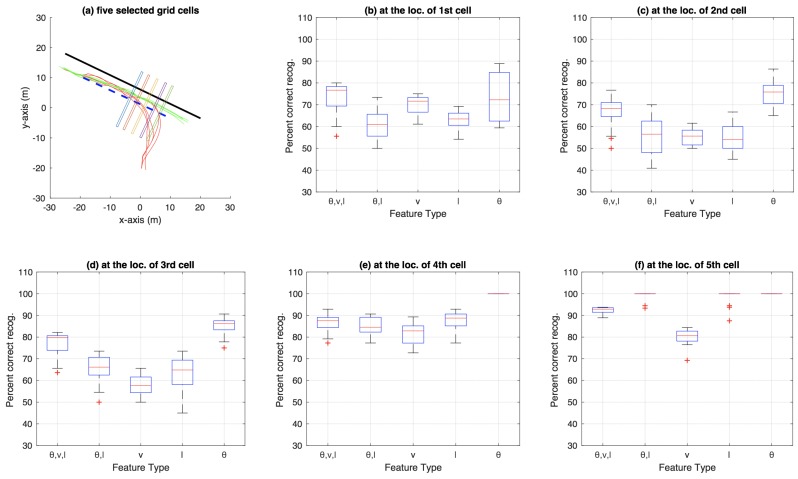
Aggregate results for car-turning dataset using particle filtering: (**a**) the five grid cells show locations where 60%, 70%, 80%, 90%, and 99% of the total car path length (for the path discretised using the grid shown in Figure 1b) has been traversed; and (**b**)–(**f**) box plots at each of the five grid-cell locations in (**a**). Each subfigure (**b**)–(**f**) shows a box plot for percentage of correct category recognition for different feature types used including (θ,v,l), (θ,l), *v*, *l* and θ.

Figure 7 shows the aggregate results on the bicycle dataset that were compiled using an n-fold approach (by varying *n* from 2 to 20, and also conducting a leave-one-out experiment) on the trajectories available in the dataset. Similarly, Figure 8 shows aggregate results for the car-turning dataset (for *n* varying from 2 to 17, as the dataset consists of 17 trajectories from each category). The figures show the percentage of correct recognition, for different set of attribute combinations as features, at five chosen grid cell locations. The five grid cell locations correspond to 0.6, 0.7, 0.8, 0.9, and 0.99 of the total path length traversed in terms of the discretisation grid.

**Figure 9:**
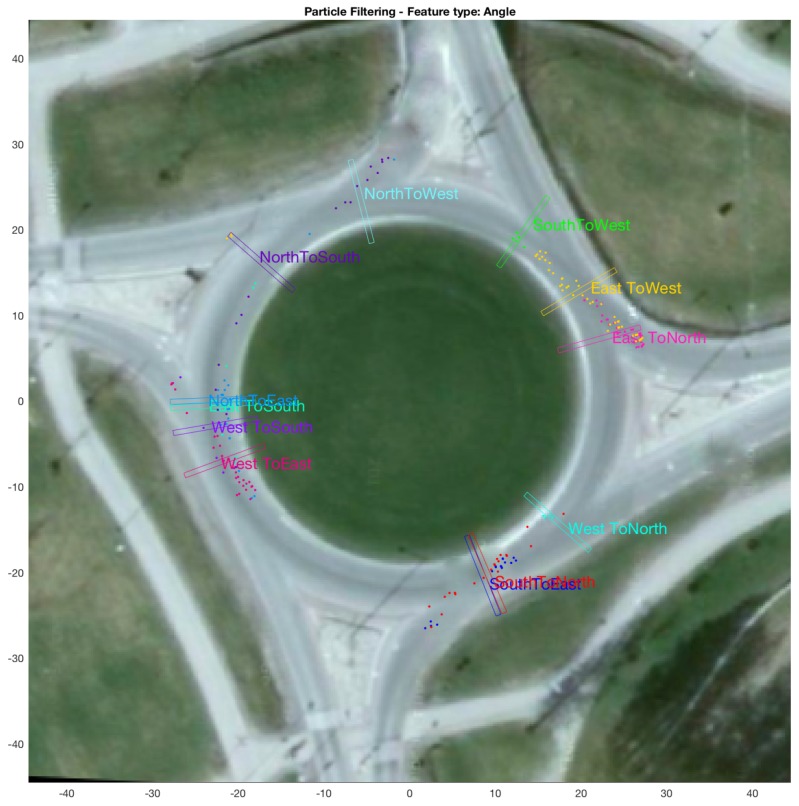
Aggregate results for roundabout dataset using particle filtering. The figure shows (colour-coded for each of the 12 test categories) the locations of the points beyond which 100% correct intention estimation was achieved for each test trajectory (plotted as coloured dots). The figure also shows (for each of the 12 test categories) the location of grid cell closest to the mean location of the points belonging to that category beyond which 100% correct intention estimation was achieved.

A high number of categories in the roundabout dataset (i.e., 16 compared to only 2 for bicycle and car-turning) demand more in-depth aggregate results compared to box plots used for the other two datasets. Therefore, the aggregate results for this dataset are presented as: (i) a confusion matrix between predicted and ground-truth exit in Table 1; and (ii) a figure showing the locations (as well as the grid-cell closest to the mean location) of points beyond which all predictions for a category were 100% correct in Figure 9. Both (i.e., the figure and the table) use θ as feature.

Each column in Table 1 represents the percentage of predictions indicating one of the four exits (i.e., the four rows) for test trajectories having the respective column direction (i.e., East, North, West or South) as the ground-truth exit. Input from each test trajectory corresponding to a column was normalised by the number of predictions made for the test trajectory (which varies, depending on the vehicle taking first, second, third, or even fourth exit after entering the round about). Each columns was then divided by the number of test trajectories that contributed to the column. The mean correct recognition for this case, i.e., the average of the correct recognition percentages for the four exits, was 75.94% using heading angle θ only as feature. Mean correct percentages for other features types were 69.24%, 69.20%, 64.58% and 66.97% for feature types (θ,v,l), (θ,l), *v* and *l*, respectively.

### 3.4. Aggregate Results Using Decision Trees

**Figure 10:**
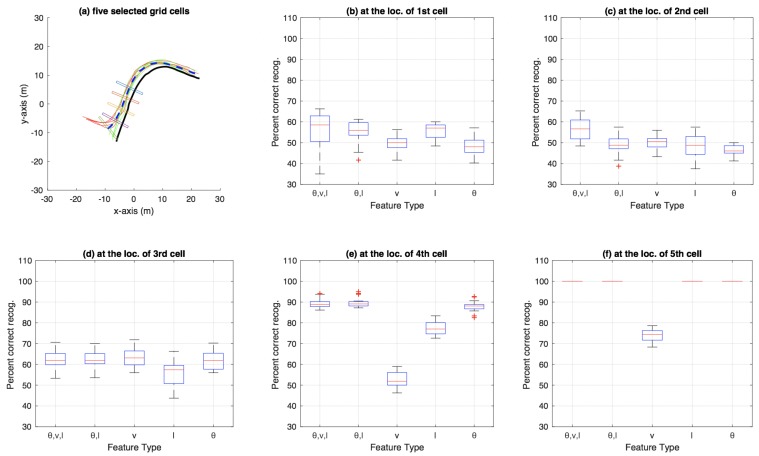
Aggregate results for bicycle dataset using decision trees.

**Figure 11:**
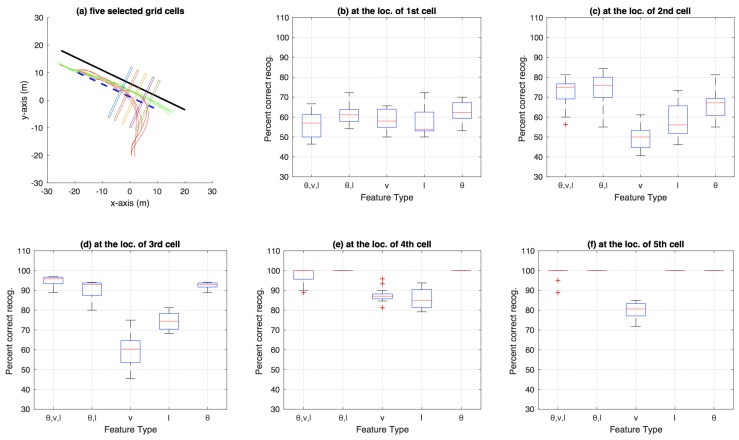
Aggregate results for car-turning dataset using decision trees.

**Figure 12:**
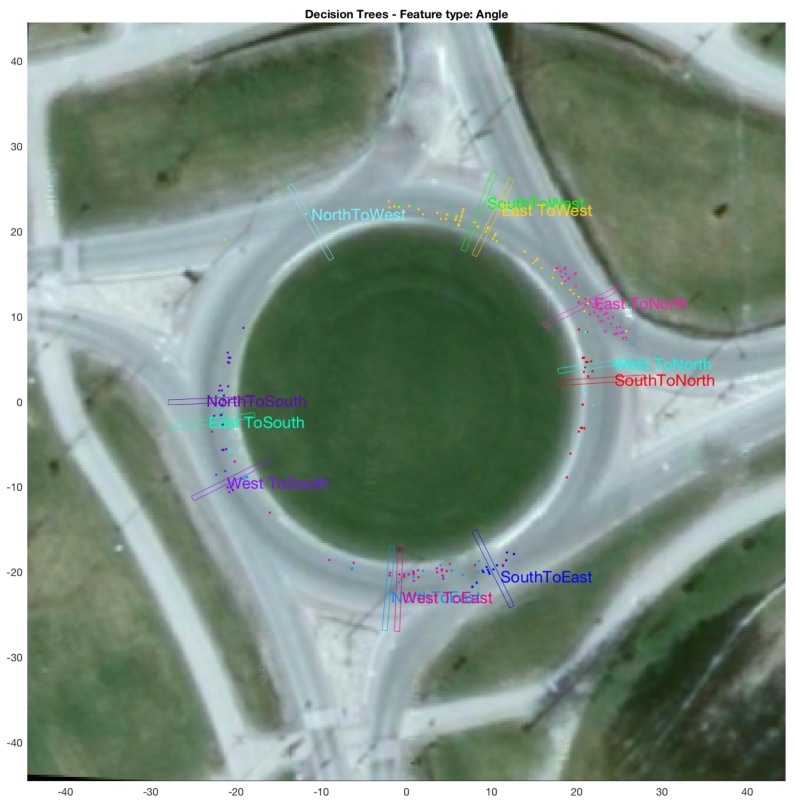
Aggregate results for roundabout dataset using decision trees. The figure shows (colour-coded for each of the 12 test categories) the locations of the points beyond which 100% correct intention estimation was achieved for each test trajectory (plotted as coloured dots). The figure also shows (for each of the 12 test categories) the location of grid cell closest to the mean location of the points belonging to that category beyond which 100% correct intention estimation was achieved.

The aggregate results on bicycle data using n-fold testing (varying *n* from 2 to 20, and also including a leave-one-out experiment) using the decision-tree based approach are presented in Figure 10. Similarly, Figure 11 shows aggregate results for the car-turning dataset (for *n* varying from 2 to 17). The results for roundabout data are presented as a confusion matrix between predicted and ground-truth exits (cf. Table 2) as well as a figure showing locations for each category beyond which all predictions made for the test trajectory in question were 100% correct (cf. Figure 12), both using θ as feature. The confusion matrix indicates an average 79.73% correct recognition (i.e., average of the four diagonal elements of the confusion matrix). The mean correct intention recognition percentage values for other feature types were 77.51%, 80.18%, 60.51% and 74.12% for the feature types of (θ,v,l), (θ,l), *v* and *l*, respectively.

### 3.5. Results Comparing Particle-Filer and Decision-Tree Methods on Roundabout Dataset

Figure 9 and Figure 12 show aggregate results for the roundabout dataset, when θ is used as feature. The figures show locations, as well as the grid cells closest to the mean location, of points beyond which all predictions made for each test category were 100% correct. From the figures, it can be observed that the locations of the shown grid cells for each category differ for the cases of particle filtering and decision trees. Table 3 shows the the difference in location of these grid cells (in terms of distance in m). For these results, the mean distance (weighted by the number of test trajectories in each category) by which particle-filter based method leads compared to decision-tree based method is 14.88 m.

Here, it is worth mentioning that Table 1 and Table 2 present confusion matrices (between predicted and ground-truth exits) for all predictions made during testing (i.e., including predictions made for each test trajectory, right from the moment of entry into the roundabout until the exit), whereas Figure 9 and Figure 12 and the table comparing the two figures, i.e., Table 3, correspond to the locations beyond which (for each test trajectory) all the predictions made are 100% correct.

## 4. Discussion

### 4.1. Speed as an Attribute

In the aggregate results presented in Figure 7, Figure 8, Figure 10 and Figure 11, it is obvious that only speed *v* as an attribute is not as discriminative as θ and *l* alone, or multiple attributes in different combination. Most obvious indication of this fact can be observed in the aggregate results for the 99% trajectory covered (cf. Figure 7f, Figure 8f, Figure 10f and Figure 11f) where, despite the two classes of trajectories in each dataset being very discriminative, speed alone fails to achieve as high recognition rates as other feature types. This makes intuitive sense because, for example in the case of bicycle riding, the riders might take a bigger curved path while turning instead of reducing their speed, especially when the physical space allows and the path is not crowded by other pedestrians and bicycles. Speed being less discriminative compared to other feature types holds for experiments on car-turning dataset as well, but a comparison of the four figures mentioned above reveals that the difference in correct recognition, for *v* against other feature types, in the case of car-turning dataset is less significant than bicycles. This also makes intuitive sense because cars on a road have to adhere to lanes more strictly, and thus reduce speeds more significantly while turning compared to bicycles on a bicycle track. These two phenomena (i.e., varying ones speed while turning) is also observable when Figure 3e and Figure 4e are compared. From the comparison, it can be observed that, in the last part of the trajectories, the speeds from the two categories are much better separated in case of cars than in the case of bicycles.

For roundabout dataset, the performance of speed as an attribute also turns out to be weak (compared to the other attributes). As mentioned in Section 3.3, different feature types, using the particle-filtering method, gave the following mean correct recognition rates: 69.24%, 69.20%, 64.58%, 66.97% and 75.94% (for the feature types of (θ,v,l), (θ,l), *v*, *l* and θ respectively). Similarly, for decision-tree based method, the mean correct recognition values as mentioned in Section 3.4 were 77.51%, 80.18%, 60.51%, 74.12% and 79.73%, respectively. These results show that for both methods, using speed as the only feature attribute yielded the minimum correct percentages i.e., 64.58% and 60.51% (for particle-filter and decision-tree bases methods, respectively). Gradient of speed i.e., acceleration was also tested as an attribute, but yielded mean correct recognition percentages of 55.99% and 58.51% (for particle-filter and decision-tree bases methods, respectively), further indicating that the speed and its derivative does not contain as useful information as other feature attributes such as angle θ and lateral position offset *l*.

### 4.2. Performance of θ, l and (θ, v, l) as Feature

**Figure 13:**
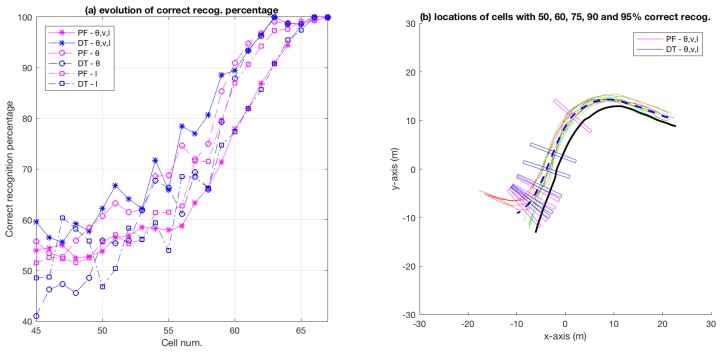
Evolution of correct recognition percentage along the bicycle track.

Figure 13 shows the evolution of correct category recognition for the bicycle dataset. Figure 13a shows how the correct-recognition percentages evolve for three different feature sets (for both the particle-filer and decision-tree based techniques), and Figure 13b shows the locations beyond which the correct recognition rate remains above 50%, 60%, 75%, 90% and 95% for both the techniques (but only for the feature vector containing all three attributes, i.e., θ, *v* and *l*).

In Figure 13a, it can be observed that, overall, decision-tree based approach with all three attributes as feature performs the best. This is apparent from the blue-asterisks curve, which in general tends to remain above the other curves. In terms of the particle-filter approach, the closest (to the decision-tree best performer) overall best performer appears to be the case where only θ is used as feature (magenta-circles curve). A comparison of the best performers for the decision-tree and particle-filter approaches indicates that the decision-tree one leads the particle-filter one by around three cells (or 1.8 m where each cell has a width of 0.6 m) for a binary-class problem such as intention estimation in the bicycle dataset.

### 4.3. Cell Size

**Figure 14:**
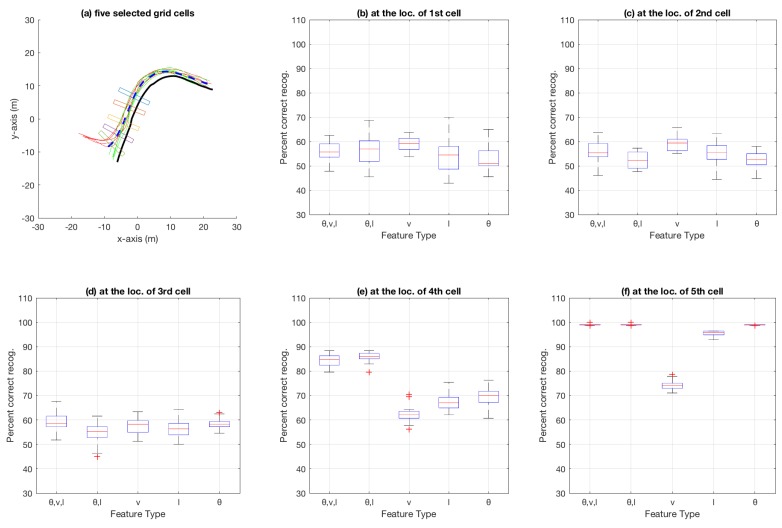
Aggregate results on bicycle dataset using decision trees, with cell width *w* = 1.2 m.

**Figure 15:**
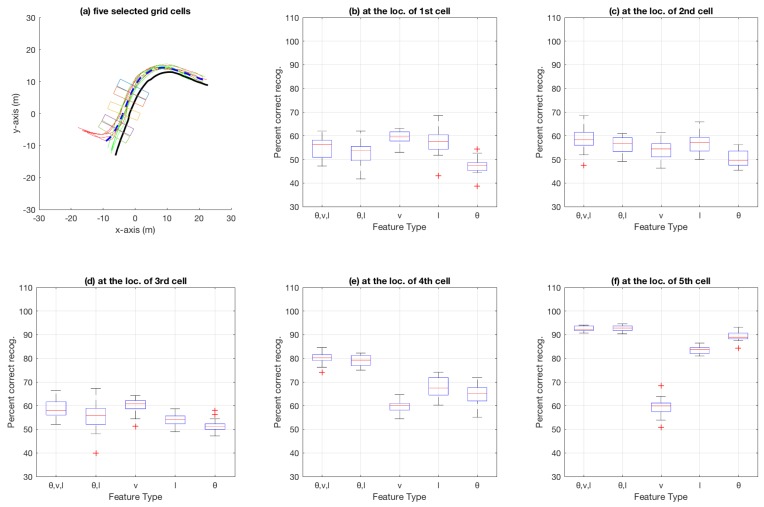
Aggregate results on bicycle dataset using decision trees, with cell width *w* = 2.4 m.

To investigate the effect of cell-size on correct category recognition, the decision-tree based experiments (the same n-fold and leave-one-out tests on the bicycle dataset as mentioned in Section 3.4) was repeated by increasing the cell-width two folds, twice—i.e., with cell widths equal to 1.2 m and 2.4 m, respectively. The aggregate results are presented in Figure 14 and Figure 15, respectively. The overall trend in the figures (i.e., comparing Figure 10, Figure 14 and Figure 15) shows that the performance (correct recognition percentage at different locations along the path) decreases with increase in cell size. This trend can be the result of increased fuzziness and decreased discrimination between the trajectory segments falling inside a cell, as the cell width increases, thus causing the trained decision tree for the respective cell to be less representative of the discriminatory characteristics of the two categories compared to a more localised, smaller, cell width.

### 4.4. Incorporating History vs. Local Snapshots

**Figure 16:**
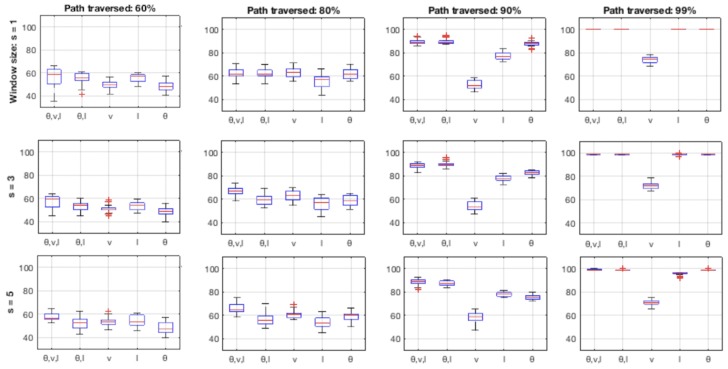
Aggregate results on bicycle dataset using decision trees with history voting over a sliding window—three rows of the subplots (from top to bottom) correspond to window sizes of 1, 3 and 5, and each of the four columns (from left to right) correspond to 60%, 80%, 90% and 99% of the total path length traversed (cf. Figure 10).

To investigate the effect and utility of trajectory history in decision-tree based approach from a different approach than increasing the cell width (as is the case in Section 4.3), another experiment was performed with a sliding window of *s* cells and a vote of category recognition results output by the individual cells inside the sliding window. Another rationale for this experiment was to study the effects of incorporation history into the decision-tree approach, which (i.e., incorporation of history) on the other hand is an inherent characteristic of algorithms such as particle filtering. Figure 16 presents the results of this experiment.

The figure shows the aggregate results for decision-tree approach on bicycle data, for sliding window size *s* equal to 1, 3 and 5 cells. The three rows of subfigures in the figure correspond to the varying *s*, and the four columns correspond to the four of the locations along the bicycle path used in earlier experiments (cf. Figure 10). The experiment indicates that employing such a history vote with small values of *s*, i.e., 3, helps reduce the variance in the correct recognition percentages, especially along the parts of bicycle path which are less discriminative in general (cf. subplots in first column of the figure, which correspond to 60% of the bicycle path traveled).

Applying history vote on the roundabout dataset using decision-tree based intention estimation showed significant increase in overall accuracy of the system. Employing a window of s=3 (corresponding to a physical distance of 1.8 m, as each grid cell is 0.6 m wide) increased the mean percentages from 77.51%, 80.18%, 60.51% and 74.12% and 79.73% (for the feature types of (θ,v,l), (θ,l), *v*, *l* and θ, respectively) to 79.21%, 82.05%, 62.92%, 75.89% and 82.71%, respectively. For s=5 (corresponding to a physical distance of 3 m), the mean correct percentages increased to 80.11%, 83.15%, 63.82%, 77.16% and 84.18%, respectively.

### 4.5. Overall Performance of Particle-Filter and Decision-Tree Based Methods

For a binary-class problem, as mentioned in Section 4.2, the decision-tree based intention estimation performs better than the particle-filter method. A closer inspection of Figure 13b shows that particle-filter methods performs better in the beginning, i.e., for locations where 50% correct recognition is achieved, and the decision-tree based method later catches up and eventually performs better than the particle-filter method (in terms of achieving higher percentages of correct recognition sooner). On a multi-class problem such as the roundabout (with 12 test categories), particle-filter performs significantly better on average (in terms of locations beyond which 100% correct recognition is achieved) than the decision-tree based method, as demonstrated by Figure 9 and Figure 12, and Table 3.

### 4.6. Utility and Applications of Intention Estimation

As mentioned in Section 1, autonomous robotic systems, be it an AGV in a warehouse or a driverless car, cannot operate in a smooth and optimal way using only an emergency breaking layer for safety. Such systems need to move smoothly, anticipating the intentions of other agents that are present in their environment well in advance (i.e., before the emergency-brake safety system needs to be triggered). In the case of roundabout data (i.e., Figure 12), if an autonomous vehicle is approaching the roundabout from South, the sooner it can anticipate the intention of a vehicle that is already inside the roundabout to be exiting at South or not exiting (and thus continuing towards the East or North exits), the better the autonomous vehicle can plan a smooth and safe manoeuvre to enter the roundabout. Such intention estimation can be even more useful for instance while estimating how to slow down for cyclists turning towards a cycle and pedestrian crossing on a road on which an autonomous car is traveling. In this way, intention estimation can endow the autonomous robotic systems to plan smooth and comfortable manoeuvres while reducing the wear and tear without compromising on safety.

Table 1 and Table 2 show that, overall, the intention of a vehicle can be predicted using the proposed particle-filter and decision-tree based method (respectively) with a high accuracy. It is worth mentioning the percentages mentioned in the tables are based on all the predictions made, as long as a vehicle remains inside the roundabout, which includes predictions made just after a vehicle enters the roundabout—the predictions which are not intuitively expected to be as reliable as the ones made when a vehicle is nearing an exit. Both tables show relatively higher level of confusion between the ground-truth and predicted exit in the case of ground-truth exit South (fourth column of the table) between predictions made in favour of South and East exits. Table 4 shows an expansion of Table 2 (Column 4) based on entry direction of test trajectories. The table shows that the trajectories initiating from North (exiting at South) contribute to this high confusion with East.

## 5. Conclusions

This paper presents a method to model behaviour of agents (such as manually driven vehicles, pedestrians, etc., that operate in the vicinity of autonomous mobile robots) that is based on a set of trajectories navigated by the agents. The paper also presents two methods (based on decision trees and particle filtering, respectively) that employ the proposed behaviour model to estimate the intention of an agent. The experiments performed using three datasets, consisting of real-world bicycle and car trajectories, validate the proposed behaviour modelling and intention estimation techniques.

The results show that the decision-tree based technique works better than the particle-filtering based technique for a binary-class problem, whereas the particle-filter based technique performs better in a multi-class problem such as a roundabout, where it, on average, achieves correct intention estimation 14.88 m earlier than the decision-tree based method. The results also show that taking a history vote on the predictions made for the anticipated future position of an agent for the decision-tree based method (which unlike particle-filters, do not take history into account inherently) improves the successful intention estimation. Among the several attributes such as heading angle θ, speed *v* and lateral position offset *l*, and combinations of those, θ seems to be the most significant attribute for the purpose of agent intention estimation, and *v* seems to be the least discriminative. In the future, we intend to investigate the suitability of the proposed methods for perception data acquired using an in-situ robot perception sensor, rather than an infrastructural-support sensor such as the ones employed for acquiring datasets used in this study.

## Figures and Tables

**Table 1:** Confusion matrix for predicted vs. ground-truth exits, using particle-filtering method for intention estimation.

	Ground-Truth Exit	East	North	West	South
Predicted Exit					
East (entering from N, W, S)		76.25	5.76	1.78	26.94
North (entering from E, W, S)		9.59	80.30	15.05	3.81
West (entering from E, N, S)		4.52	13.71	82.80	4.81
South (entering from E, N, W)		9.64	0.23	0.35	64.42

**Table 2:** Confusion matrix for predicted vs. ground-truth exits, using decision-tree method for intention estimation.

	Ground-Truth Exit	East	North	West	South
Predicted Exit					
East (entering from N, W, S)		78.34	4.23	1.88	21.46
North (entering from E, W, S)		10.99	88.53	12.42	1.01
West (entering from E, N, S)		0.79	6.71	77.78	3.24
South (entering from E, N, W)		9.87	0.53	7.92	74.29

**Table 3:** Method and distance, leading (i.e., succeeding earlier in) intention estimation in terms of the grid-cell location closest to the mean decision point for each test category. PF represents particle-filter based method, and S–E represent South–East.

Category	S-E	S-N	S-W	E-N	E-W	E-S	N-W	N-S	N-E	W-S	W-E	W-N
Leading method	PF	PF	PF	PF	PF	PF	PF	PF	PF	PF	PF	PF
Leading by (m)	1.8	28.2	5.4	4.2	13.2	1.8	6	17.4	33	6.6	27	19.8

**Table 4:** Expansion of Table 2 (Column 4) based on entry direction of test trajectories—values in percent.

	Entry Direction (for an Eventual Exit at South)	East	North	West
Predicted Exit				
(Number of test trajectories)		(5)	(30)	(5)
East		10.94	23.33	20.74
North		5.26	0	2.86
West		25.95	0	0
South		57.84	76.67	76.41

## References

[1] Brooks R. The Big Problem with Self-Driving Cars Is People. IEEE Spectr..

[2] Kwak J.Y., Ko B.C., Nam J.Y. (2017). Pedestrian intention prediction based on dynamic fuzzy automata for vehicle driving at nighttime. Infrared Phys. Technol..

[3] Lidström K., Larsson T. Model-based Estimation of Driver Intentions Using Particle Filtering. Proceedings of the 11th International IEEE Conference on Intelligent Transportation Systems.

[4] Lidström K., Larsson T. Act normal: Using uncertainty about driver intentions as a warning criterion. Proceedings of the 16th World Congress on Intelligent Transportation Systems.

[5] Li S., Wang W., Mo Z., Zhao D. (2018). Cluster Naturalistic Driving Encounters Using Deep Unsupervised Learning. arXiv.

[6] Liebner M., Baumann M., Klanner F., Stiller C. Driver intent inference at urban intersections using the intelligent driver model. Proceedings of the IEEE Intelligent Vehicles Symposium.

[7] Martinez C.M., Heucke M., Wang F.Y., Gao B., Cao D. (2018). Driving Style Recognition for Intelligent Vehicle Control and Advanced Driver Assistance: A Survey. IEEE Trans. Intell. Transp. Syst..

[8] Jain A., Koppula H.S., Raghavan B., Soh S., Saxena A. Car that Knows Before You Do: Anticipating Maneuvers via Learning Temporal Driving Models. Proceedings of the IEEE International Conference on Computer Vision.

[9] Maghsood R., Johannesson P. (2016). Detection of steering events based on vehicle logging data using hidden Markov models. Int. J. Veh. Des..

[10] Okamoto K., Berntorp K., Cairano S.D. (2017). Driver Intention-based Vehicle Threat Assessment using Random Forests and Particle Filtering. Int. Fed. Autom. Control.

[11] Bokare P.S., Maurya A.K. Acceleration-Deceleration Behaviour of Various Vehicle Types. Proceedings of the World Conference on Transport Research.

[12] Maurya A.K., Bokare P.S. (2012). Study of deceleration behaviour of different vehicle types. Int. J. Traffic Transp. Eng..

[13] Alonso J.D., Vidal E.R., Rotter A., Muhlenberg M. (2008). Lane-Change Decision Aid System Based on Motion-Driven Vehicle Tracking. IEEE Trans. Veh. Technol..

[14] Sivaraman S., Morris B., Trivedi M. Learning multi-lane trajectories using vehicle-based vision. Proceedings of the IEEE International Conference on Computer Vision Workshop.

[15] Dong C., Dolan J.M., Litkouhi B. Intention estimation for ramp merging control in autonomous driving. Proceedings of the IEEE Intelligent Vehicles Symposium.

[16] Kucner T., Saarinen J., Magnusson M., Lilienthal A.J. Conditional transition maps: Learning motion patterns in dynamic environments. Proceedings of the IEEE International Conference on Intelligent Robots and Systems.

[17] Tango F., Botta M., Serra R., Cucchiara R. (2009). ML Techniques for the Classification of Car-Following Maneuver. AI*IA 2009: Emergent Perspectives in Artificial Intelligence.

[18] Salomonson I., Rathai K.M.M. (2015). Mixed Driver Intention Estimation and Path Prediction Using Vehicle Motion and Road Structure Information. Master’s Thesis.

[19] Zhao M., Kathner D., Jipp M., Soffker D., Lemmer K. Modeling Driver Behavior at Roundabouts: Results from a Field Study. Proceedings of the IEEE Intelligent Vehicles Symposium.

[20] Zhao M., Kathner D., Soffker D., Jipp M., Lemmer K. Modeling Driving Behavior at Roundabouts: Impact of Roundabout Layout and Surrounding Traffic on Driving Behavior. https://core.ac.uk/download/pdf/84275712.pdf.

[21] Sivaraman S., Trivedi M.M. (2013). Looking at Vehicles on the Road: A Survey of Vision-Based Vehicle Detection, Tracking, and Behavior Analysis. IEEE Trans. Intell. Transp. Syst..

[22] Cappé O., Godsill S.J., Moulines E. (2007). An overview of existing methods and recent advances in sequential Monte Carlo. Proc. IEEE.

[23] Thrun S., Fox D., Burgard W., Dellaert F. (2001). Robust Monte Carlo lozalization for mobile robots. Artif. Intell..

[24] Wolf J., Burgard W., Burkhardt H. (2005). Robust Vision-Based Localization by Combining an Image Retreival System with Monte Carlo Localization. IEEE Trans. Robot..

[25] Arulampalam M.S., Maskell S., Gordon N., Clapp T. (2002). A tutorial on particle filters for online nonlinear/non-Gaussian Bayesian tracking. IEEE Trans. Signal Process..

[26] Doucet A., Johansen A.M. (2011). A Tutorial on Particle Filtering and sMoothing: Fifteen Years Later.

[27] Li T., Bolic M., Djuric P.M. (2015). Resampling Methods for Particle Filtering: Classification, implementation, and strategies. IEEE Signal Process. Mag..

[28] Mitchell T. (1997). Machine Learning.

[29] Ho T.K. Random Decision Forests. Proceedings of the International Conference on Document Analysis and Recognition.

[30] Wang Y., Xiu C., Zhang X., Yang D. (2018). WiFi Indoor Localization with CSI Fingerprinting-Based Random Forest. Sensors.

[31] Verikas A., Gelzinis A., Bacauskiene M. (2011). Mining data with random forests: A survey and results of new tests. Pattern Recognit..

[32] Viscando Traffic Systems AB, Sweden. https://viscando.com/.

[33] Fan H., Kucner T.P., Magnusson M., Li T., Lilienthal A.J. (2018). A Dual PHD Filter for Effective Occupancy Filtering in a Highly Dynamic Environment. IEEE Trans. Intell. Transp. Syst..

